# Self‐expandable tubular collagen implants

**DOI:** 10.1002/term.2685

**Published:** 2018-05-15

**Authors:** Luuk R.M. Versteegden, Marja ter Meer, Roger M.L.M. Lomme, J. Adam van der Vliet, Leo J. Schultze Kool, Toin H. van Kuppevelt, Willeke F. Daamen

**Affiliations:** ^1^ Department of Biochemistry, Radboud Institute for Molecular Life Sciences Radboud university medical center Nijmegen The Netherlands; ^2^ Department of Radiology and Nuclear Medicine Radboud university medical center Nijmegen The Netherlands; ^3^ Department of Surgery Radboud university medical center Nijmegen The Netherlands

**Keywords:** biodegradable implant, biomaterial, collagen, hollow organs, regenerative medicine, self‐expandable, tissue engineering, vascular

## Abstract

Collagen has been extensively used as a biomaterial, yet for tubular organ repair, synthetic polymers or metals (e.g., stents) are typically used. In this study, we report a novel type of tubular implant solely consisting of type I collagen, suitable to self‐expand in case of minimal invasive implantation. Potential benefits of this collagen scaffold over conventional materials include improved endothelialization, biodegradation over time, and possibilities to add bioactive components to the scaffold, such as anticoagulants. Implants were prepared by compression of porous scaffolds consisting of fibrillar type I collagen (1.0–2.0% (*w*/*v*)). By applying carbodiimide cross‐linking to the compressed scaffolds in their opened position, entropy‐driven shape memory was induced. The scaffolds were subsequently crimped and dried around a guidewire. Upon exposure to water, crimped scaffolds deployed within 15–60 s (depending on the collagen concentration used), thereby returning to the original opened form. The scaffolds were cytocompatible as assessed by cell culture with human primary vascular endothelial and smooth muscle cells. Compression force required to compress the open scaffolds increased with collagen content from 16 to 32 mN for 1.0% to 2.0% (*w*/*v*) collagen scaffolds. In conclusion, we report the first self‐expandable tubular implant consisting of solely type I collagen that may have potential as a biological vascular implant.

## 1 INTRODUCTION

The main function of tubular structures, such as blood vessels or bile ducts, is the transportation of fluids. These structures may become blocked due to atherosclerotic diseases (e.g., stenoses) or malignancies. Biomaterials have proven to be effective tools to ensure patency of tubular structures. Currently, the majority of devices for clinical use are permanent implants, mostly metals or medical textiles such as expanded polytetrafluoroethylene. The concept of biodegradability has gained interest over the last years as it comes with obvious benefits. The absence of foreign body material at long‐term follow‐up may reduce the risk of complications such as occlusion of blood vessels and infection (Tenekecioglu et al., 2016). Currently used biodegradable implants for tubular tissues, often prepared from synthetic polymers or magnesium, only play a supportive role in keeping the structure open but do not actively facilitate re‐endothelialization. Materials of biological origin such as type I collagen may be more suitable as they are well known for their excellent biocompatibility (Glowacki & Mizuno, 2008).

Another important feature for tubular implants from biological origin would be the ability to self‐expand (shape memory). Biodegradable thermally induced shape memory tubular implants prepared from synthetic polymers have been developed, and the Igaki‐Tamai PLLA‐based coronary stent has been successfully evaluated in humans (Nishio et al., 2012). However, alternatives prepared from materials of only natural origin are lacking.

Recently, a novel method was developed to endow collagen implants with entropy‐driven shape memory using straightforward carbodiimide cross‐linking in combination with compression techniques (Versteegden et al., 2016). The method was used to create a star‐shaped tubular scaffold with a shape memory designed for luminal closure (Versteegden et al., 2017). In the current communication, we report the construction of a novel tubular implant consisting of only type I collagen, with an inverse shape memory. This scaffold has the ability to self‐expand from a crimped state to a tubular shape, similar to a self‐expandable stent used in vascular disease treatment. Parameters such as deployment time, cytocompatibility, and mechanical compression strength are addressed. To the best of our knowledge, this is the first self‐expanding scaffold in the field of vascular medicine that consists of solely type I collagen and which may be suitable for minimally invasive implantation procedures, that is, it does not require the use of open surgery with accompanying risks of infection (Koens et al., 2015).

**Figure 1:**
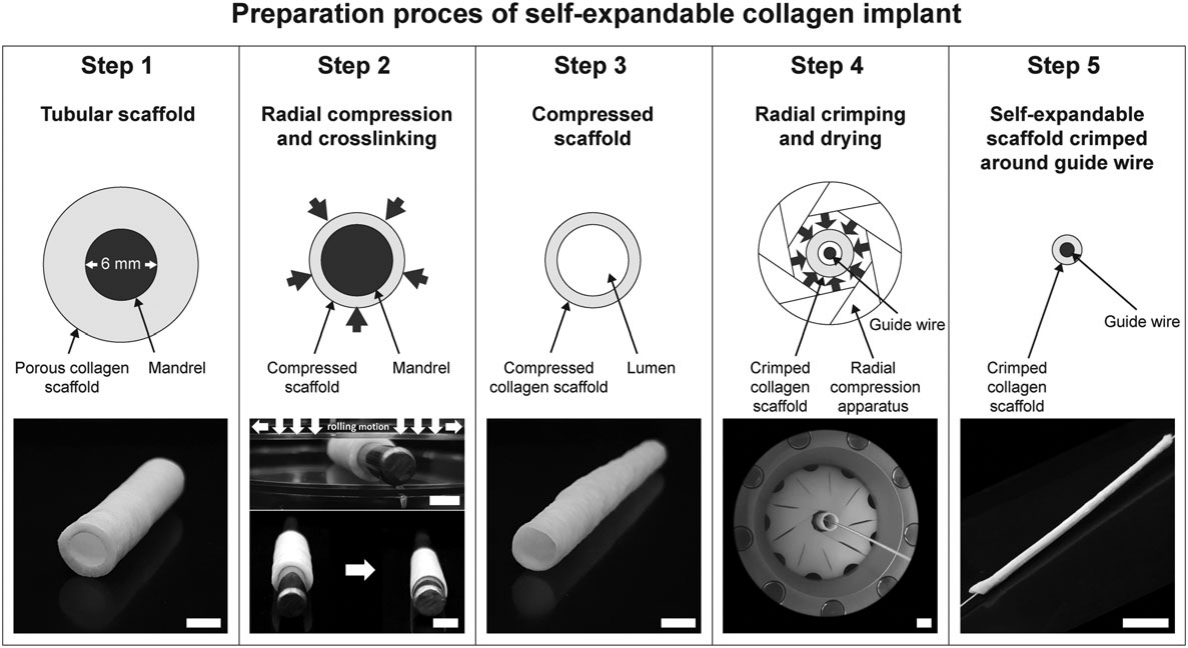
Production process of the self‐expandable vascular implant. Step 1: Porous tubular collagen scaffolds with a luminal diameter of 6 mm (or 4 mm, not shown) were used as starting point. Step 2: The scaffolds were manually compressed between two flat objects and chemically cross‐linked with the mandrel present. Step 3: Compression and cross‐linking resulted in a collagen scaffold with a film‐like appearance. This scaffold was used for mechanical testing. Step 4: The compressed scaffold was crimped around a metal guide wire using an automated compression machine and air‐dried in crimped position. Step 5: The dry crimped collagen scaffold around a guide wire in crimped position that can expand upon exposure to water. Scale bar = 6 mm

Tubular collagen scaffolds with a luminal diameter of 4 and 6 mm were prepared from insoluble fibrillar type I collagen purified from bovine Achilles tendon by swelling the collagen (1.0%, 1.5%, and 2.0% *w*/*v*) in 0.25M acetic acid followed by a casting, molding, freezing, and lyophilization process (Pieper, Oosterhof, Dijkstra, Veerkamp, & van Kuppevelt, 1999; Versteegden et al., 2016). These tubular scaffolds (Figure 1, Step 1) were used as the starting point for preparation of the self‐expandable collagen scaffolds. Scaffolds were compressed between two aluminum objects by applying pressure under a rolling motion until the scaffold had a film‐like appearance (Figure 1, Step 2). Next, carbodiimide cross‐linking was applied to stabilize the construct in the tubular film‐like shape that it should have after implantation in a blood vessel. This step is crucial in inducing shape memory. For cross‐linking, the scaffolds were incubated in 50mM 2‐morpholineoethane sulphonic acid (MES buffer, pH 5.0, USB, Ohio, USA) containing 40% (*v*/v) ethanol, 33mM N‐ethyl‐3‐(3‐dimethylaminopropyl) carbodiimide hydrochloride (EDC, Merck Schuchardt OHG, Hohenbrunn, Germany), and 6mM N‐hydroxysuccinimide (NHS, Fluka Chemie AG, Buchs, Switserland), followed by subsequent washing steps with 0.1M Na_2_HPO_4_ (2 × 1 hr), 1.0M NaCl (2 × 15 min), 2.0M NaCl (2 × 15 min), and demineralized water (6 × 15 min) after which they were placed in 70% (v/v) ethanol (Figure 1, Step 3). Subsequently, tubular scaffolds were placed over a metal guidewire, crimped with 500 kPa using an automated radial compression machine (Blockwise Engineering, Tempe, AZ, USA) and air‐dried for 15 min (Figure 1, Steps 4 + 5).

To visualize the self‐expanding property, the crimped scaffolds with original diameters of 6 mm were placed in a plastic tube with an internal diameter of 7 mm using a metal guide wire. Water was pumped through the tube using a 50‐ml syringe to allow scaffolds to expand. In this experiment, collagen scaffolds made from suspensions containing 1.0%, 1.5%, and 2.0% (*w*/*v*) collagen were analysed. Videos and images were recorded using a Sony HDR‐CX405 camera (Sony, Tokyo, Japan). To show that the self‐expandability also works in the presence of blood plasma, the 1% scaffolds were also exposed to human blood plasma, obtained from a healthy volunteer.

**Figure 2:**
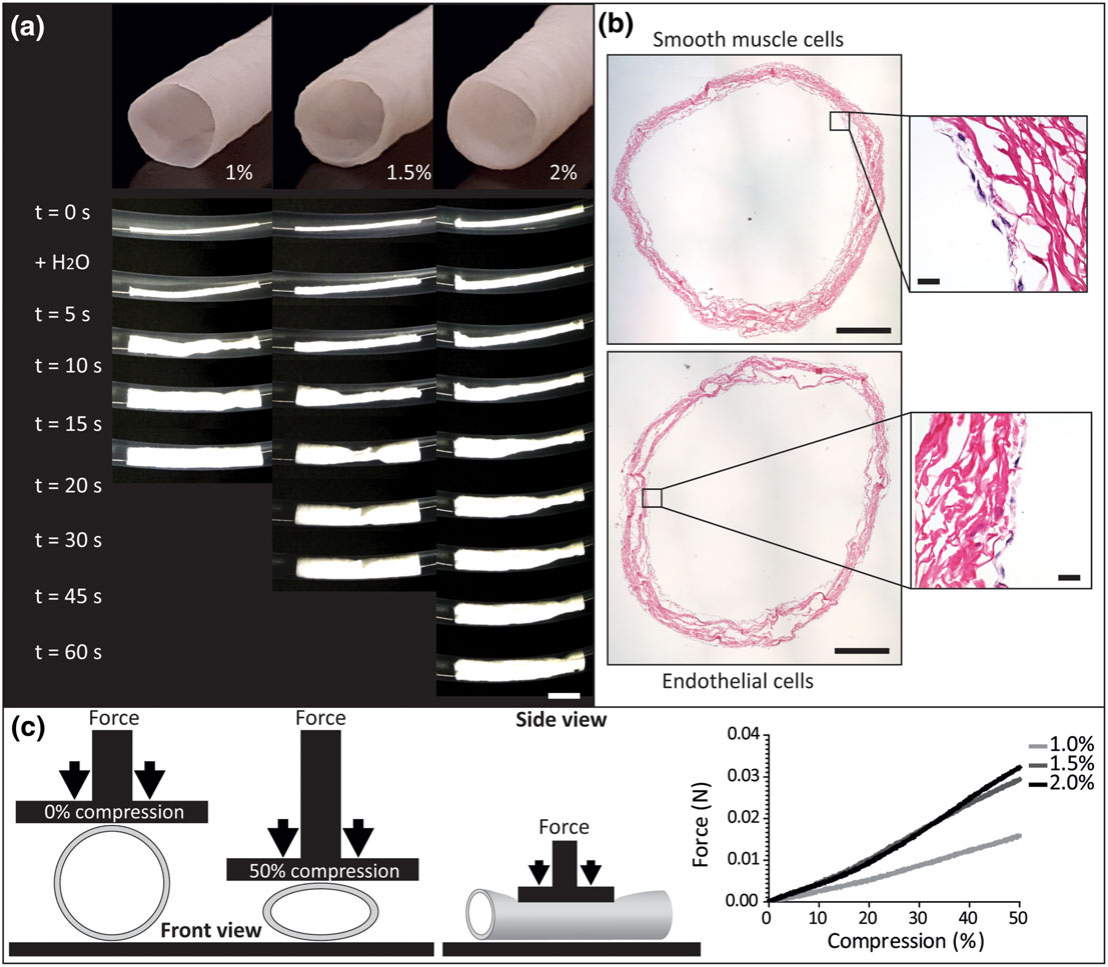
Scaffold deployment and cytocompatibility and mechanical characterization. (a) The crimped tubular collagen scaffolds of 1.0, 1.5, and 2.0% (*w*/*v*) were exposed to water inside a plastic tube. All scaffolds deployed within 1 min, a higher collagen content resulted in a longer deployment time, scale bar is 1 cm. (b) Overview of endothelial cells and smooth muscle cells seeded on 1% collagen scaffolds with enlargements. Scale bars are 1 mm and 10 μm. (c) Schematic representation of the mechanical analysis (left) and the results depicted in a graph (right) showing the force needed for compression of the tubes [Colour figure can be viewed at http://wileyonlinelibrary.com]

To assess the ability of the tubular scaffolds to withstand compressive force, a tensile tester (Zwick/Roell Z2.5, Ulm, Germany) was used to apply force using a 20 mm square block as depicted in Figure 2c. This test simulated local compression of the scaffolds, and subsequently, the ability of the scaffolds to return to their original diameters was observed. A strain rate of 10 mm/min was applied until the scaffolds (6 mm diameter, 60 mm length, 1.0%, 1.5%, and 2.0% (*w*/*v*) collagen) were compressed to 50% of their original diameter. A self‐expandable metal (nitinol) stent with comparable dimensions was similarly compressed to make a rough comparison of compression force between our scaffolds and existing devices. Applied force and displacement were recorded.

Cytocompatiblity of three scaffolds (4 mm diameter, 1.0%, 1.5%, and 2.0% (w/v) collagen) was evaluated by a cell culture experiment with human primary endothelial cells and human smooth muscle cells (Sciencecell, Carlsbad, CA, USA). In brief, scaffolds were cut to a length of 1 cm, placed in 1.5‐ml Eppendorf tubes, and washed with 70% (*v*/v) ethanol for disinfection. Next, scaffolds were washed extensively with phosphate buffered saline (pH 7.4) after which 1‐ml cell suspension in endothelial growth medium‐2 (Lonza, Basel, Switzerland) or smooth muscle cell medium (Sciencecell, Carlsbad, CA, USA) containing 1.3 × 10^5^ cells was added. Seeded scaffolds were cultured overnight under rotation at 10 rpm in a 37 °C incubator. After 24 hr, scaffolds were transferred to 6‐wells plates, cultured for 6 days, fixed with 4% paraformaldehyde in phosphate buffered saline, and embedded in paraffin. Sections (4 μm) were stained with haematoxylin/eosin using fluoroshield mounting medium (Sigma, St Louis, MO, USA) and analysed using a Leica DMLB microscope (H&E).

A self‐expandable tubular implant was developed using a simple method based on chemical cross‐linking of collagen, combined with compression and subsequent crimping of the scaffold. Upon exposure to water, the scaffold instantly deployed and fixed to the tube wall, after which the metal guide wire could be easily removed (Movie S1, 1.0% (*w*/*v*) collagen scaffold). Deployment time increased with a higher collagen content as the 1.0% collagen scaffolds fully deployed within 15 s, followed by within 25 and 60 s for the 1.5% and 2.0% scaffolds, respectively (Figure 2a). The 1.0% scaffolds were also tested upon exposure to human blood plasma, where they fully expanded in 45 s.

When submerged in water, the collagen scaffolds fully expand, and after mechanical compression in water, the scaffolds always returned to their fully expanded state. Compression strength of the expanded collagen scaffolds increased with increasing collagen content: 16, 29, and 32 mN for the 1.0, 1.5, and 2.0% (*w*/*v*) scaffolds, respectively (Figure 2c). For the 1.5 and 2.0% (w/v) construct, significantly more force is required for 50% compression (*p* < .001, one‐way ANOVA with Bonferroni post hoc test). However, compared with the self‐expandable nitinol stent, the collagen scaffolds are relatively weak at this stage, as a force of 1 N was required for 50% compression of the nitinol stent. The cytocompatibility study showed that both cell types covered the majority of the surface on which they were seeded after 6 days of culturing (Figure 2b). H&E staining showed that endothelial cells displayed an endothelial cell‐like morphology. Note that due to the applied seeding procedure, both endothelial and smooth muscle cells were present on both the luminal side and the outside of the scaffold but did not penetrate into the interior of the scaffold as shown by H&E (Figure 2b, zoomed views). The scaffold shown in Figure 2 is a 1.0% (*w*/*v*) collagen scaffold that was also representative for the 1.5% and 2.0% (w/v) as those scaffolds also showed a confluent lining. Overall, these results indicate appropriate cytocompatibility.

The collagen‐based, tubular‐shaped implant described can be crimped or folded to a fraction of its original size but will re‐expand upon exposure to water or blood plasma. This makes it a suitable material for minimally invasive implantation techniques. Vascular medicine was chosen as an example as the use of self‐expandable devices is common practice in patient care in the treatment of vascular injuries in acute (e.g., vessel perforation) and semiacute (e.g., pseudoaneurysm) situations (Jeong, 2016). Vascular stents and endografts are inserted through endovascular sheaths and expanded on delivery, contact with liquids can be avoided until the construct is at the correct location. For secure positioning without displacement in a vessel with blood flow, rapid expansion is essential and it is therefore advantageous that the scaffold deploys in less than 1 min and presses firmly against the vessel wall to ensure maintenance of blood flow and to minimize the risk of migration of the device after implantation (Xue, Dai, & Li, 2010).

A common limitation of vascular implants is the lack of re‐endothelialization, thus the inability of endothelial cells to grow onto the implant. The observation that endothelial cells covered a large part of the luminal surface is promising in this respect. Cells did not migrate into the wall of the implant. This may be especially relevant for smooth muscle cells that may impair vascular healing due to excessive proliferation. The observation that these cells could not enter the scaffold indicates that the collagen may provide a temporary barrier that may help to prevent intimal hyperplasia and restenosis (Inoue et al., 2011). In addition, the use of flexible collagen scaffolds may circumvent a common limitation of metal vascular implants that is the occurrence of stress fractures due to their rigidity, restricting their use in bending points (i.e., groin or knee).

The shape‐memory principle, which is driven by newly established hydrophobic interactions upon cross‐linking (Versteegden et al., 2016), may not only be relevant for treatment of vascular damage, but can also be applied for a number of other organs such as bile ducts (Blero, Huberty, & Deviere, 2015). In combination with modern imaging techniques, a personalized 3D‐printed mold for the collagen scaffold may be created for any desired shape and application.

The main limitation of the current tubular‐shaped scaffolds for both vascular and other applications is the relatively poor compression strength. Increasing collagen content in the suspension improved the compression strength. However, scaffold strength can only be increased up to a certain limit using this method. Collagen content above 2% complicates the scaffold production process because of the high viscosity of the collagen suspension. Whether this is a problem in vivo in the field of vascular medicine remains to be assessed: Human veins also compress easily yet are successfully used as arterial conduits (Wilasrusmee et al., 2017). In case of vascular implants, the stiffness of the stent must be large enough to resist migration; therefore, these scaffolds require fortification and optimization for in vivo use. Additional methods to reinforce the collagen scaffold include the use of synthetic biodegradable polymers, or (biodegradable) metal meshes.

Collagen is often applied as a biomaterial because of its biodegradability and biocompatibility, but it has also been associated with thrombogenicity in blood vessel applications. Gelatin (denatured collagen), however, has been used to impregnate Dacron vascular grafts used in clinical practice, so as to make the material less permeable. This coating was not associated with increased thrombosis or restenosis (Chou et al., 2017). When thrombogenicity presents as a problem, the material can be coated with heparin to prevent blood clotting using the same carbodiimide cross‐linking step applied in this study (Lammers, van de Westerlo, Versteeg, van Kuppevelt, & Daamen, 2011).

In this short communication, we report the first self‐expandable tubular implant consisting of solely type I collagen. It may allow implantation using minimal invasive techniques as it deploys within 60 s and is cytocompatible for vascular endothelial and smooth muscle cells. The implant may have potential for use as a biological vascular implant.

## CONFLICTS OF INTEREST

The authors have declared that there is no conflict of interest.

## Supporting information


**Movie S1**. Example of a 1.0% (w/v) scaffold expanding upon contact with water.
